# Mechanisms of Cotranslational Protein Maturation in Bacteria

**DOI:** 10.3389/fmolb.2021.689755

**Published:** 2021-05-25

**Authors:** Jiří Koubek, Jaro Schmitt, Carla Veronica Galmozzi, Günter Kramer

**Affiliations:** Center for Molecular Biology of Heidelberg University (ZMBH) and German Cancer Research Center (DKFZ), DKFZ-ZMBH Alliance, Heidelberg, Germany

**Keywords:** protein folding, chaperone recognition, nascent chain processing, cotranslational assembly, ribosomal exit tunnel, trigger factor, DnaK

## Abstract

Growing cells invest a significant part of their biosynthetic capacity into the production of proteins. To become functional, newly-synthesized proteins must be N-terminally processed, folded and often translocated to other cellular compartments. A general strategy is to integrate these protein maturation processes with translation, by cotranslationally engaging processing enzymes, chaperones and targeting factors with the nascent polypeptide. Precise coordination of all factors involved is critical for the efficiency and accuracy of protein synthesis and cellular homeostasis. This review provides an overview of the current knowledge on cotranslational protein maturation, with a focus on the production of cytosolic proteins in bacteria. We describe the role of the ribosome and the chaperone network in protein folding and how the dynamic interplay of all cotranslationally acting factors guides the sequence of cotranslational events. Finally, we discuss recent data demonstrating the coupling of protein synthesis with the assembly of protein complexes and end with a brief discussion of outstanding questions and emerging concepts in the field of cotranslational protein maturation.

## Introduction

Rapidly growing bacterial cells contain between 20,000 and 70,000 ribosomes (Liveris et al., 1991; Bremer and Dennis, 2008) that actively translate mRNA to duplicate the proteome and enable generation times below 30 min in optimal conditions. Bacterial ribosomes translate at a rate of about 15–20 codons per second, synthesizing several proteins per minute. Nearly all newly synthesized proteins are enzymatically processed at their N-terminus. Furthermore, cytosolic proteins must fold to reach their native structure, often with the help of chaperones, while proteins destined for the cell envelope must be recognized, targeted and translocated into or across the cytoplasmic membrane. Considering the dynamics of translation, these decisions need to be made in a timely and robust manner. These maturation steps are coupled with protein synthesis and guided by several maturation factors that dynamically engage the polypeptide, starting when the N-terminus emerges from the ribosomal exit tunnel and ending only after the newly synthesized protein has been released by translation termination. The ribosome constitutes an integral component of all cotranslational maturation steps by providing a unique folding environment inside the ribosomal exit tunnel and near the ribosomal surface, guiding the folding process by translating mRNAs with a protein-specific rhythm and by serving as a docking site for the coordinated engagement of chaperones, processing and targeting factors.

Here we report on recent advances in the understanding of cotranslational protein maturation focusing on protein folding and assembly in the bacterial model system *Escherichia coli* (*E. coli*). We describe the cellular machineries involved and how their function is integrated with translation to create a highly versatile protein surveillance system that can maintain the integrity of the complex proteome. We would like to refer to other excellent, recent reviews on related topics, including a review providing a detailed description of the energetics of protein folding on the ribosome (Waudby et al., 2019), the role of the ribosome in protein folding (Cassaignau et al., 2020; Liutkute et al., 2020b), the role of translation speed (Samatova et al., 2021), and mechanisms of protein maturation in prokaryotes and eukaryotes (Kramer et al., 2019).

## The Ribosome as the Platform for Protein Maturation

Genetically encoded proteins are produced by ribosomes, large ribonucleoproteins composed of two subunits that are highly conserved in all domains of life. Ribosomes not only catalyze the formation of the peptide bond but also provide a unique folding environment for nascent proteins. In *E. coli*, the small ribosomal subunit (30S) is composed of the 16S rRNA and 22 ribosomal proteins, while the large ribosomal subunit (50S) consists of the 5S and the 23S rRNA and 33 proteins. Decoding the mRNA occurs within the 30S subunit, and the respective amino acid is added to the peptide chain by the action of the peptidyl transfer center (PTC) in the 50S subunit. The growing chain traverses the large subunit through the ribosomal exit tunnel, which is 80–100 Å long and can accommodate a linear polypeptide of approximately 30 residues. The width of the exit tunnel is not uniform but contains a 10 Å constriction formed by extensions of the ribosomal proteins uL22 and uL4 about 30 Å downstream of the PTC as well as the vestibule, a 20 Å widening close to the tunnel exit (Ban et al., 2000; Voss et al., 2006).

**FIGURE 1:**
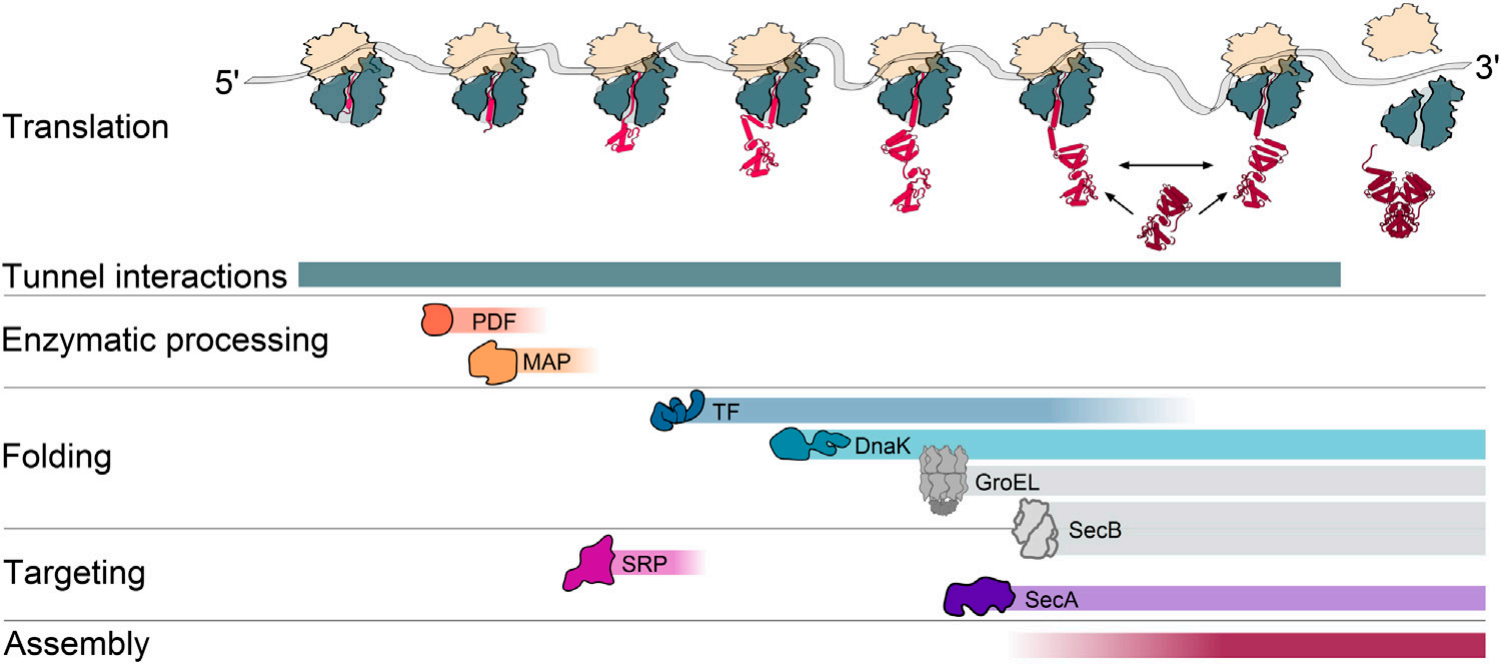
A cascade of cotranslational processes guides nascent chain maturation. During translation, the nascent chain undergoes a series of interactions that contribute to maturation. These can include interactions with the ribosomal exit tunnel, sequential interactions with peptide deformylase (PDF) and methionine aminopeptidase (MAP) for deformylation and methionine excision at the N-terminus, interactions with the cotranslationally acting chaperones trigger factor (TF), the Hsp70 DnaK, interactions with the signal recognition particle (SRP) and the protein translocation ATPase SecA for targeting to the inner membrane, as well as interactions with another nascent or fully synthesized subunit for the assembly of protein complexes. Colored gradients indicate when during translation the interactions generally occur. A prevalent cotranslational action of the Hsp60 chaperonin GroEL and the protein export chaperone SecB is not yet fully established and therefore depicted in gray.

The different steps of protein maturation are coordinated with the stage of protein synthesis to ensure that the right factor meets the right target at the right time (Figure 1). Several maturation factors bind in the vicinity of the tunnel exit, often by interacting with the ribosomal protein uL23 that is located on the ribosomal surface but also reaches inside the ribosomal tunnel (Kramer et al., 2002; Buskiewicz et al., 2004; Huber et al., 2011). The first step is the enzymatic processing of the N-terminus (Sandikci et al., 2013) that must be completed before the cell makes a triage decision on whether the ribosome-bound nascent chain is destined for the cytoplasm or translocation. The signal recognition particle (SRP), binds and targets nascent inner membrane proteins (IMPs) to the translocon, while proteins that are translocated across the membrane to the periplasmic space or the outer membrane are engaged by the SecA ATPase and sometimes also the protein export chaperone SecB (Saraogi and Shan, 2014). Cytoplasmic proteins may be sequentially engaged by chaperones generally starting with Trigger Factor (TF) (Merz et al., 2008; Lakshmipathy et al., 2010). Further co- and post-translational folding steps may include other chaperones, including DnaK, GroEL, and SecB (Hartl and Hayer-Hartl, 2002; Castanie-Cornet et al., 2014). Some nascent subunits engage other subunits for cotranslational assembly of protein complexes (Shieh et al., 2015), thus also coupling the last step of protein maturation to translation.

### Enzymatic Processing of Nascent Chains by PDF and MAP

The first residue of nascent bacterial proteins is N-formyl-methionine. However, mature proteins generally lack formylation and often also the N-terminal methionine (Giglione et al., 2015). The formyl group is removed by the peptide deformylase (PDF) which is the rate-limiting prerequisite for further methionine excision by the methionine aminopeptidase (MAP) (Yang et al., 2019). Both of these enzymes bind near the exit of the ribosomal tunnel (Bingel-Erlenmeyer et al., 2008; Sandikci et al., 2013) and while an excess of one factor reduces the binding of the other, a recent structural study suggested that MAP may reposition itself to a secondary binding site if excess of PDF is present (Bhakta et al., 2019).

N-terminal processing is the essential, first maturation step of nascent chains. Retaining the formylated methionine appears to destabilize the protein, by serving as a potent degron (Piatkov et al., 2015; Kim et al., 2018) for protein quality control. Early processing of nascent chains is critical, as PDF and MAP activity is influenced by other ribosome-associated factors, such as TF and SRP (Sandikci et al., 2013; Bornemann et al., 2014) (Figure 1). Enzymatic assays with purified proteins and synthesized peptides showed that PDF has only very loose specificity requirements for the residues that follow the N-terminal N-formylmethionine (Hu et al., 1999), whereas MAP disfavors N-termini with certain amino acids at the penultimate position (Xiao et al., 2010). However, the relatively slow reaction kinetics in these *in vitro* assays could not explain how the majority of proteins in an actively translating cell are processed in time (Yang et al., 2019). *In vivo*, the presence of the ribosome accelerates the reaction kinetics by 2–4 orders of magnitude (Yang et al., 2019), achieving high levels of processing within the few seconds between the emergence of the N-terminus from the tunnel and engagement of other partitioning factors, like TF or SRP.

The nascent chain can be deformylated and the methionine can be cleaved off as soon as 45 amino acids are synthesized, with the peak of activity on 70 amino acids-long nascent chains and a decrease of activity for nascent chains longer than 100 amino acids (Sandikci et al., 2013; Ranjan et al., 2017; Yang et al., 2019). This length dependence might be imposed by three factors: 1) enhanced binding of SRP and TF to longer nascent chains which outcompete PDF and MAP; 2) the positioning of the active sites of PDF and MAP on the ribosome may favor interactions with short nascent chains; and 3) limited mobility and accessibility of longer N-termini due to secondary or tertiary structure formation. Consequently, transmembrane domains (TMDs) of membrane proteins that may fold within the ribosomal exit tunnel and comprise an N-out topology could sometimes escape post-translational processing by PDF and retain the formyl group (Ranjan et al., 2017).

Even for substrates with the optimal processing length, the deformylation rates varied by two orders of magnitude. The lowest rates were observed for the nascent chain of HemK that can fold within the ribosomal exit tunnel (Mercier and Rodnina, 2018) and inner membrane protein LepB (Ranjan et al., 2017). The deformylation rate of LepB but not HemK was further inhibited by the presence of SRP. TF, in contrast, did not affect the reaction as it generally binds nascent chains longer than 100 amino acids (Oh et al., 2011). The deformylation of shorter nascent chains of another inner membrane protein, FtsQ, was only weakly influenced by SRP (Yang et al., 2019). This difference might be due to the greater distance of the TMD from the N-terminus (Yang et al., 2019), which could grant PDF an extended time window to act on nascent FtsQ, before the emergence of the first TMD triggers SRP engagement. The excision of the N-terminal methionine of an optimal MAP substrate and of shorter suboptimal substrate is not influenced by the presence of TF and SRP. In contrast, longer nascent chains with a suboptimal penultimate residue are less efficiently processed by MAP in the presence of TF or SRP.

### First Folding Steps of Nascent Chains Inside the Ribosomal Tunnel

The ribosomal exit tunnel shields the early nascent chain from the environment. Although it is narrow, some folding steps can occur in its interior. Initial folding may include the formation of helices between the PTC and the constriction site (Woolhead et al., 2004; Agirrezabala et al., 2017; Su et al., 2017). As such helices are unlikely to pass through the narrow constriction site as translation continues, this compaction may be transient and not relevant for native folding. Multiple studies reported on helix formation beyond the constriction site (Lu and Deutsch, 2005; Bhushan et al., 2010; Tu and Deutsch, 2010; Lin et al., 2012; Agirrezabala et al., 2017; Su et al., 2017). This includes short alanine-based peptides with high helical propensity also in solution (Marqusee et al., 1989; Lu and Deutsch, 2005; Bhushan et al., 2010; Lin et al., 2012), as well as peptides that may dynamically alternate between helical and extended conformations, including hydrophobic transmembrane helices (Bano-Polo et al., 2018).

The emergence of hydrophobic helices constitutes a signal for membrane targeting, either by recruiting SRP for the cotranslational targeting of IMPs (Saraogi and Shan, 2014; Schibich et al., 2016) or SecA, that binds translating ribosomes to cotranslationally initiate protein translocation across the membrane (Huber et al., 2011; Huber et al., 2017). Accordingly, cleavable N-terminal signal sequences (SS) of translocated proteins and transmembrane domains of IMPs are predicted to form helices inside the tunnel (Halic et al., 2006; Robinson et al., 2012), although the helical conformation may not always dominate (Lange et al., 2016). The helicity of the emerging nascent chain segment could confer a signal that prevents binding of the chaperone TF, which would compete with both targeting factors for overlapping binding sites on the ribosome. Consistent with this model, a helix inside the ribosomal exit tunnel was reported to decrease ribosome binding of TF (Lin et al., 2012). It was speculated that helix formation near the tunnel loop of uL23 may generate a signal that can be transferred to the surface exposed part of uL23, which forms the general docking site for TF, SRP and SecA (Kramer et al., 2002; Gu et al., 2003; Huber et al., 2011).

Going beyond the formation of an alpha-helical secondary structure, some small domains can also fold within the vestibule. This includes nascent chain compaction and the formation of beta-hairpins (Kosolapov and Deutsch, 2009; O'brien et al., 2010; Tu et al., 2014), as well as native folding of the zinc finger domain of ADR1 (Nilsson et al., 2015) or folding of the N-terminal domain of HemK inside the ribosomal exit tunnel (Liutkute et al., 2020a). However, the prevalence of such folding events in the tunnel is not yet clear. Interestingly, a formation of partial tertiary structures inside the vestibule was suggested to spatially cluster hydrophobic residues and facilitate TF recognition (O'brien et al., 2010). Therefore, early folding inside the ribosomal exit tunnel may be a discriminating factor for polypeptide triaging. The formation of helices inside the tunnel may indicate a TMD and facilitate cotranslational membrane insertion, while tertiary structures may indicate a newly formed core of a cytosolic protein.

### The Ribosome Guides Cotranslational Folding Outside of the Ribosomal Exit Tunnel

As the nascent chain emerges from the ribosome, the spatial constraints of the tunnel are relieved while the limiting impact of the ribosome on the conformational space of the nascent chain partially remains. Supported by studies on multiple model proteins (Hsu et al., 2007; Ellis et al., 2008; Ellis et al., 2009; Kelkar et al., 2012; Holtkamp et al., 2015; Kim et al., 2015; Koubek et al., 2017; Nilsson et al., 2017; Farias-Rico et al., 2018; Mercier and Rodnina, 2018; Kemp et al., 2019; Liutkute et al., 2020a) it is estimated that at least 30% of the cytosolic *E. coli* proteome folds independently of chaperones (Ciryam et al., 2013). Folding of these proteins is therefore solely determined by the intrinsic biophysical properties of the amino acid sequence and the influence of the ribosome. The ribosome influences the folding of the emerging polypeptide in three major ways: 1) the vectorial synthesis itself ensures a step-wise addition of new residues and folding information; 2) the varying speed of translation provides defined time windows during which folding intermediates can sample the folding landscape; and 3) the large, negatively charged ribosomal surface directly impacts nascent chain folding. Although it may be difficult to distinguish how each of the listed mechanisms contributes toward the overall efficiency of folding, multiple examples highlight the importance of the ribosome as a folding mediator.

Vectorial synthesis (Marsden et al., 2018) appears to be particularly important for the folding of larger, multi-domain proteins, for which the gradual emergence of the nascent chain prevents non-productive long-range interactions and promotes domain-wise folding (Bitran et al., 2020). Supporting this model, the folding of the small SH3 domain (Eichmann et al., 2010; Guinn et al., 2018) or the Ig domain I27 (Tian et al., 2018) follows similar trajectories on the ribosome and upon refolding *in vitro*, while the N-terminal domain of HemK folds differently in both folding scenarios. Upon emergence from the ribosome, the N-terminal domain of nascent HemK acquires an intermediate folding state within the ribosomal exit tunnel and rapidly folds into a native-like structure once the full domain has emerged. In solution, however, the HemK N-domain undergoes rapid transitions between folded and unfolded states without stable folding intermediates (Holtkamp et al., 2015; Mercier and Rodnina, 2018; Nissley and O'Brien, 2018; Kemp et al., 2019; Liutkute et al., 2020a). Similarly, the cytoskeletal protein spectrin was suggested to have differing folding pathways on and off the ribosome (Nilsson et al., 2017; Kemp et al., 2020). Finally, recent folding studies of the multi-domain protein EF-G reveal a highly intricated network of interactions to guide the folding process. Once fully emerged, the N-terminal domain folds and supports the co-translational folding of domain II (Liu et al., 2019b; Chen et al., 2020). In contrast, the central domain III of EF-G acquires a stable fold only post-translationally, upon interactions with folded C-terminal parts of the protein (Liu et al., 2019a).

Ribosome profiling has shown that the translation rate not only varies between transcripts but also during translation of a single transcript (Ingolia et al., 2009; Oh et al., 2011). These translation speed alterations provide time windows for nascent chains to sample their folding landscape (O'brien et al., 2014a; O'brien et al., 2014b). Studies on the relationship between translation kinetics and protein folding revealed a correlation between the accumulation of rare codons, conferring slow translation due to the lower abundance of their cognate tRNAs, and the predicted formation of folding intermediates or domains (Clarke and Clark, 2008; Jacobs and Shakhnovich, 2017). The concept that codon usage may guide folding is supported by experimental evidence. For example, supplementation of additional tRNAs that decode rare codons clustered in the *E. coli* gene *sufI* led to increased protease susceptibility of the SufI nascent chains, indicating altered cotranslational folding (Zhang et al., 2009). Similarly, the replacement of rare codons in the human CFTR genes with optimal codons causes increased aggregation *in vitro* (Kim et al., 2015) and silent mutations of the *cat* gene in *E. coli* resulted in the synthesis of a protease-susceptible chloramphenicol acetyltransferase and decreased fitness in chloramphenicol-containing media (Walsh et al., 2020).

There is initial evidence for a retrograde transfer of information from the nascent chain to the ribosome to influence translation speed. Examples are proteins containing ribosome arrest peptides. Most of the currently described arrest peptides are utilized to regulate gene expression or play a role in eukaryotic quality control mechanisms (Joazeiro, 2017). One prominent model peptide is the *E. coli* SecM protein that can stall its own synthesis due to complex interactions between the arrest sequence and the ribosomal exit tunnel (Nakatogawa and Ito, 2001; Zhang et al., 2015). SecM controls the expression of the *secA* gene that is positioned downstream of *secM* within the same operon and translated from the same mRNA (Nakatogawa and Ito, 2001). Additional examples include the membrane protein insertion and folding monitor MifM from *Bacillus subtilis* (Chiba and Ito, 2012) and peptides that can sense the presence of specific small molecules such as erythromycin, chloramphenicol, tryptophan, arginine, S-adenosyl-methionine or polyamine [reviewed in (Ito and Chiba, 2013)]. Besides dedicated arrest peptides, stretches of positively charged residues can also interact with the negatively charged tunnel wall and reduce translation speed (Charneski and Hurst, 2013) or cause ribosomal stalling (Chandrasekaran et al., 2019).

A feedback loop between the nascent chain and the ribosome may also confer a speed-up of translation. Series of experiments using stalling sequences as force sensors (Ismail et al., 2012; Goldman et al., 2015; Marino et al., 2016; Kemp et al., 2020) have demonstrated that cotranslational folding can resolve translation arrests conferred by the arrest peptide of SecM. Considering the high frequency of stalling motifs in the genome (for example the stalling motif PPX is not underrepresented in the genome) (Ito and Chiba, 2013; Peil et al., 2013; Woolstenhulme et al., 2013), translation pauses may constitute a frequent autoregulatory mechanism to guide cotranslational protein folding: A translational pause may provide enough time for nascent proteins to compact into a folding intermediate, and this folding could generate a pulling force on the nascent chain which allows translation to resume. A detailed study analyzing how stalling sites are distributed in the genome and how conserved they are between species may further support the existence and importance of such a mechanism. Suggesting that translation slowdown can also confer misfolding, a recent study exploring the folding of nascent calerythrin showed that stalled chains can quickly adopt a misfolded conformation, while ongoing translation confers a kinetic barrier for misfolding (Alexander et al., 2019).

The negatively charged surface of the ribosome can delay the folding of a polypeptide chain that is close to its surface (Kaiser et al., 2011; Kelkar et al., 2012) but also trigger misfolding (Alexander et al., 2019). The basis of this activity is that the ribosome can destabilize the structure of the nascent chain by 1–2 kcal/mol (Samelson et al., 2016; Waudby et al., 2018), regardless of whether this structure represents a folded or misfolded state (Liu et al., 2017). By lowering the energetic barrier, the ribosome allows more efficient sampling of possible conformations, helping to avoid kinetic traps. The destabilization effect of the ribosomal surface on a particular domain structure decreases with ongoing translation. The impact of ribosome proximity on folding varies between nascent chains but is generally reduced 45–55 residues away from the PTC (Cabrita et al., 2016; Samelson et al., 2016). How folding is impacted by ribosomes is not entirely clear but probably involves direct interactions of ribosome-proximal residues with the ribosomal surface (Hsu et al., 2009). This interaction may be diminished by charge repulsion between negatively charged nascent chain residues and the negatively charged ribosomal surface (Knight et al., 2013), possibly leading to a delay in folding (Farias-Rico et al., 2018).

## Folding Support by Chaperones

### Trigger Factor is the First Chaperone that Engages Nascent Chains

TF is the only known chaperone that binds bacterial ribosomes and, according to this privileged position, the first chaperone that interacts with nascent chains (Kramer et al., 2002; Hoffmann et al., 2010; Gloge et al., 2014; Balchin et al., 2016). TF was discovered as a soluble factor required for the folding and translocation of pro-OmpA (Crooke and Wickner, 1987). TF ablation is not lethal and does not detectably reduce the growth rate of *E. coli* under normal growth conditions; but enhances the sensitivity of mutants to certain antibiotics or detergents (Teter et al., 1999; Oh et al., 2011) and induces a mild heat shock response (Deuerling et al., 2003). Analyses of TF function revealed that TF binds a broad spectrum of nascent chains to support folding (Deuerling et al., 1999; Hoffmann et al., 2010; Oh et al., 2011). Suggested by the findings that TF prevents the aggregation and assists the refolding of some proteins *in vitro,* TF may have additional, ribosome-independent chaperone activities (Huang et al., 2000; Maier et al., 2001). TF exists in three-state equilibrium with around one-third being bound to the ribosome and two-thirds existing in monomer-dimer equilibrium in the cytosol. Monomeric TF binds to vacant ribosomes with a dissociation constant (K_d_) of 1–2 μM (Patzelt et al., 2002; Raine et al., 2006) and cycles on and off translating ribosomes with a mean residence time of 10–15 s (Maier et al., 2003; Kaiser et al., 2006; Rutkowska et al., 2008). In contrast, TF binding to polypeptides in solution in the absence of ribosomes is rather short-lived (∼100 ms) (Maier et al., 2001) with K_d_ values varying from 1 μM for unfolded proteins (Scholz et al., 1997; Maier et al., 2001) to 100 μM for short oligopeptides (Patzelt et al., 2001). *In vitro* binding studies suggested that TF preferentially binds to peptides enclosing eight amino acid short motifs enriched in aromatic and basic residues, which are frequently found in proteins (about every 30 residues), whereas peptide stretches with acidic residues are disfavored (Patzelt et al., 2001; Kaiser et al., 2006; Merz et al., 2008). Highlighting its function as a chaperone of nascent chains, TF exhibits about 10-fold elevated binding affinity for ribosome-nascent chain complexes (RNCs) than for idle ribosomes (Raine et al., 2006; Rutkowska et al., 2008). The dimeric state may constitute a storage form of TF but may also serve to encapsulate partially folded proteins and assist in the formation of larger protein complexes (Martinez-Hackert and Hendrickson, 2009).

To support nascent chain folding, the ATP-independent TF provides a large substrate interaction surface that contains multiple binding sites distributed over all three domains of TF (Saio et al., 2014): The C-terminal domain, located in the middle of the chaperone, forms two protruding helical arms and is responsible for the main chaperone function (Genevaux et al., 2004; Kramer et al., 2004a; Merz et al., 2006; Saio et al., 2014). The N-terminal domain mediates binding to the ribosomal protein uL23 (Hesterkamp et al., 1997; Kramer et al., 2002; Kristensen and Gajhede, 2003) and also contributes to substrate binding and chaperone activity (Genevaux et al., 2004; Kramer et al., 2004b; Merz et al., 2006; Saio et al., 2014). The third domain of TF, the peptidyl-prolyl isomerase (PPIase) domain, catalyzes the cis/trans isomerization of prolyl peptide bonds and accelerates prolyl isomerization-limited folding reactions (Stoller et al., 1995; Hesterkamp and Bukau, 1996). The PPIase domain also provides a binding site for unfolded proteins but is dispensable for the main chaperone function (Kramer et al., 2004a; Merz et al., 2006; Lakshmipathy et al., 2007). Studies *in vitro* implied that TF can bind to nascent chains with a length as short as 40 amino acids (Houben et al., 2005; Lakshmipathy et al., 2007; Merz et al., 2008). However, selective ribosome profiling experiments (Becker et al., 2013) revealed that *in vivo*, TF detectably binds to RNCs when nascent chains have an average length of about 100 amino acids (Oh et al., 2011).

**FIGURE 2:**
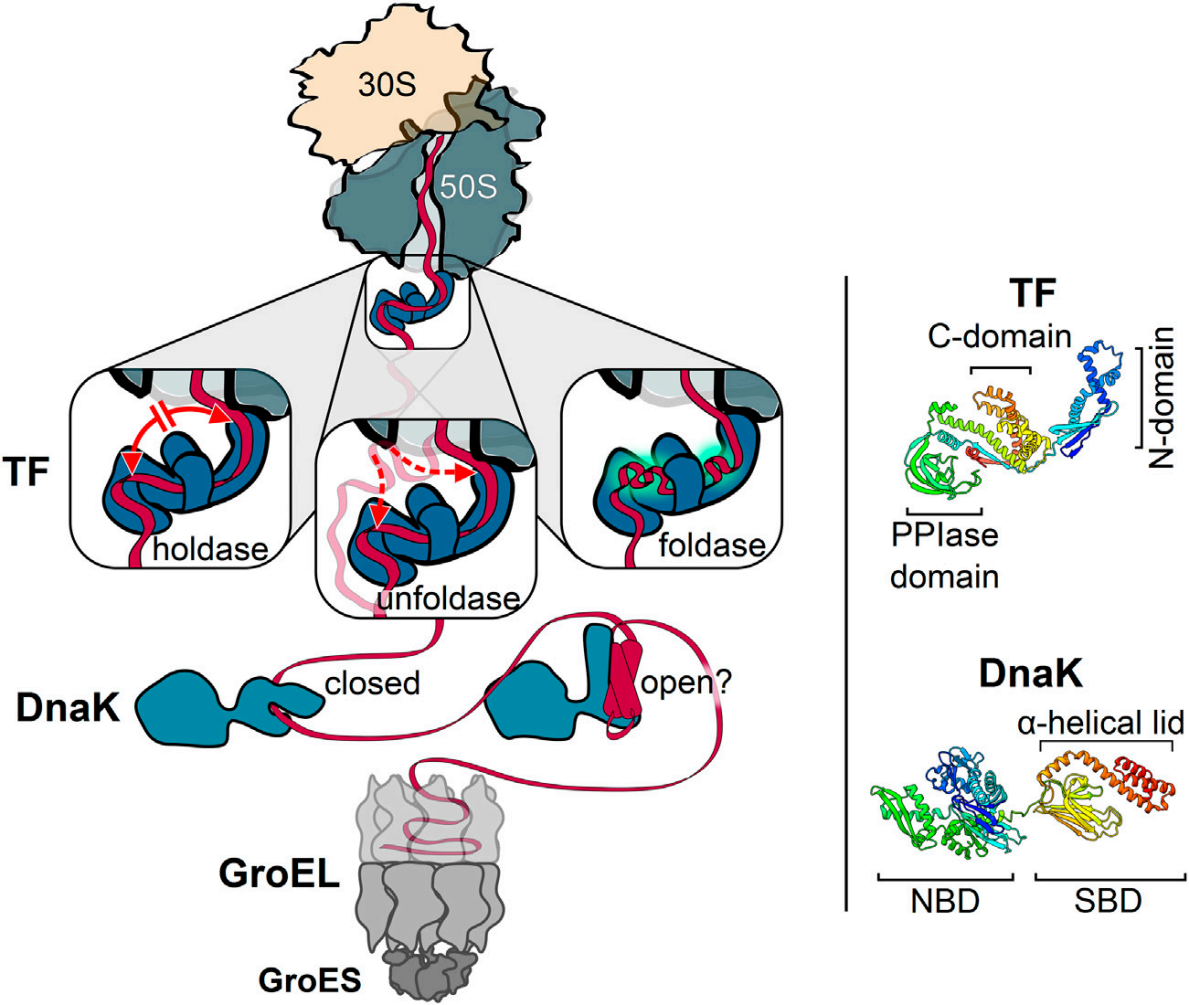
Key players of the chaperone network acting on nascent cytosolic proteins. Trigger factor (TF) is the only chaperone that binds directly to the ribosome. Depicted are the three different activities associated with TF: TF can act as a holdase by preventing long-distance interactions, thus slowing down folding **(left)**; TF can act as an unfoldase by reversing off-pathway folding events **(middle)**; TF can act as a foldase by promoting local interactions within the nascent substrate **(right)**. The bacterial Hsp70, DnaK, acts downstream of TF and employs two alternative substrate binding modes. DnaK can bind to linear **(left)** and possibly also to compacted nascent chain segments **(right)** with a closed and open substrate-binding domain (SBD), respectively. The cotranslational activity of GroEL may occur without full encapsulation of the nascent chain by GroES binding. Each of these chaperones can cycle on and off nascent chains. Ribbon diagrams of TF [PDB: 2MLX, (Saio et al., 2014)] and DnaK [PDB: 2KHO, (Bertelsen et al., 2009)] are shown on the right, colored from blue (N-terminus) to red (C-terminus) with the domains indicated. N-domain, N-terminal domain. C-domain, C-terminal domain. PPIase-domain, peptidyl-prolyl isomerase domain. NBD, nucleotide-binding domain. Molecular graphics and analyses performed with UCSF Chimera (Pettersen et al., 2004).

Employing its multi-valent substrate interaction properties, TF can exert alternative functions in cotranslational protein folding (Figure 2): 1) As a holdase, TF restricts the rate of structural rearrangements within the nascent polypeptide and thereby prevents the formation of non-native tertiary structures or inter-domain misfolding (Agashe et al., 2004; O'brien et al., 2011; Oh et al., 2011; Hoffmann et al., 2012; Saio et al., 2014; Liu et al., 2019b); 2) As a foldase, TF might enhance the efficiency of protein folding by promoting local interactions within its nascent substrates and by protecting partially folded states from distant interactions (Agashe et al., 2004; Hoffmann et al., 2012; Mashaghi et al., 2013; Singhal et al., 2015); 3) As an unfoldase, TF reverses premature folding of off-pathway folding intermediates to prevent cotranslational protein misfolding and aggregation (Hoffmann et al., 2010; Hoffmann et al., 2012; Saio et al., 2014). The unfoldase activity might be particularly important for TF’s function in the translocation of pre-secretory proteins, in conjunction with the ATPase SecA and the secretion-dedicated chaperone SecB (Castanie-Cornet et al., 2014). The folding activities of TF are most likely determined by the properties of the nascent chain and the interactions with TF. The unfoldase activity of TF is limited to loosely folded substrates, suggesting TF can revert non-productive folding of intermediates (Hoffmann et al., 2012). Extensive interactions between the nascent chain and TF with fast binding rates may promote the holdase function, while the gradual reduction of interactions due to local structure formation may guide the folding to the native state.

### DnaK Binding to Nascent Chains

The major *E. coli* Hsp70 DnaK together with its co-chaperone DnaJ and the nucleotide exchange factor GrpE constitutes an important component of the protein quality control machinery (Frydman, 2001; Mayer and Bukau, 2005). DnaK is a constitutively expressed, abundant cytosolic chaperone, and expression is further increased by several stresses including a heat-shock (Genevaux et al., 2004). DnaK is dispensable under non-stressed conditions and becomes essential in the cold and at growth temperatures above 37°C (Bukau and Walker, 1989).

DnaK activity is modulated by an allosteric mechanism that involves the N-terminal nucleotide-binding domain (NBD) and a C-terminal substrate-binding domain (SBD), which determines the affinity of DnaK for its substrates (Zhu et al., 1996; Bertelsen et al., 1999; Mayer and Kityk, 2015). ATP-bound DnaK has low substrate affinity and rapid substrate interaction kinetics. ATP hydrolysis, triggered by DnaJ and the bound substrate, induces the closing of the α-helical lid over the hydrophobic substrate-binding cleft of the SBD to stabilize the chaperone-substrate complex. The role of the Hsp40 DnaJ is to engage and deliver substrates to DnaK and to stimulate ATP hydrolysis. Substrate release is mediated by ADP dissociation and ATP binding, triggered by the nucleotide exchange factor GrpE.

DnaK contributes to all major processes that maintain cellular proteostasis, including the folding of newly synthesized polypeptides, refolding of misfolded proteins, disassembly of aggregates, degradation of proteins, disassembly of oligomeric complexes and modulation of the stability and activity of some natively folded proteins. DnaK has two distinct substrate interaction modes (Figure 2): The well-established, classical mode is that DnaK employs its SBD to bind short, extended peptide motifs with a hydrophobic core of four to five residues, enriched in leucine, isoleucine, valine, phenylalanine and tyrosine, and flanked by basic residues (Rüdiger et al., 1997; Kityk et al., 2012). This binding mode allows DnaK to engage denatured proteins by binding surface-exposed hydrophobic segments that are normally buried inside the molecule (Hartl and Hayer-Hartl, 2002; Zhao et al., 2020). In the recently described alternative interaction mode DnaK also binds compacted folding intermediates *via* the groove in the substrate-binding domain, while the lid remains partially or fully open. This mode of DnaK binding may stabilize or destabilize folding intermediates and also help to coordinate the final steps of folding (Schlecht et al., 2011; Mashaghi et al., 2016; Zhao et al., 2019).

Studies exploring the DnaK interactome in non-stressed cells revealed that DnaK binds many nascent polypeptides (Deuerling et al., 1999; Teter et al., 1999; Deuerling et al., 2003). A more recent proteome-wide study analyzing newly synthesized proteins identified more than 700 DnaK interactors (Calloni et al., 2012). It remains unclear, which of these proteins are engaged cotranslationally. The DnaK interactors generally have reduced solubility, are often lowly expressed, are enriched in large multi-domain proteins and are often part of hetero-oligomeric complexes (Tartaglia et al., 2010; Calloni et al., 2012). Together, these findings suggest that DnaK substrates are particularly vulnerable and prone to aggregation. Many of them may require the assistance of multiple chaperone systems to reach their native state, including TF and GroEL. How DnaK function is coordinated with the progress of translation, how it is coordinated with other chaperones and how the chaperones' action overlap to create functional redundancy and robustness of the network remains currently unclear.

### Possible Cotranslational GroEL Action

The Hsp60 GroEL is the only essential chaperone in *E. coli*. GroEL belongs to the group I chaperonins, large barrel-shaped complexes composed of two heptameric rings stacked back to back (Saibil et al., 2013). Each of these rings forms a cavity to bind non-native proteins ranging between 20 and 60 kDa (Ewalt et al., 1997; Houry et al., 1999; Fujiwara et al., 2010). The co-chaperone GroES acts as a lid to close the folding chamber (Hartl et al., 2011). GroEL binds substrates through hydrophobic surfaces in its apical domain and substrate folding takes place after encapsulation by GroES binding to the cis-ring (Horwich et al., 2007; Horwich et al., 2009; Castanie-Cornet et al., 2014). ATP binding to the opposite ring (trans-ring) provokes GroES dissociation and substrate release (Weissman et al., 1995).

The current model assumes that GroEL binds substrates post-translationally. Suggesting it may also engage nascent chains, two *in vitro* studies showed a nascent chain dependent GroEL association with RNCs (Ying et al., 2005; Ying et al., 2006). Cotranslational GroEL binding could be particularly important for substrates that are stringently dependent on GroEL for folding (Kerner et al., 2005; Fujiwara et al., 2010). Considering that nascent chains are C-terminally connected to the ribosome, it has been speculated that cotranslational GroEL action may be independent of GroES binding to the cis-chamber. One attractive model is that GroEL binding mainly serves to protect nascent chains from undesirable interactions or misfolding. Considering binding persists until translation terminates, the released polypeptides may be encapsulated post-translationally by GroES recruitment and fold inside the closed cavity. Alternatively, GroEL may also support folding cotranslationally, either by loose GroES binding to the cis-chamber or without closure of the hydrophobic chamber, as demonstrated before (Chaudhuri et al., 2001).

### Chaperones Collaborate to Form a Robust Protein Folding Network

The folding of thousands of structurally diverse proteins in the crowded cytosol is a considerable challenge for the cell. To achieve this task, also under conditions of stress, TF, DnaK, and GroEL together form a network of chaperones that synergistically act in the folding process. Although each individual chaperone has a different mechanism of action, the robustness of the network benefits from significant redundancy. Supporting the overlapping function of chaperones, the ablation of TF can be efficiently balanced by a mild overexpression of DnaK and an about two-fold elevated association of DnaK with nascent chains (Teter et al., 1999). The loss of DnaK alone has only a moderate impact on cell viability under non-stress conditions (Bukau and Walker, 1989). Revealing the cooperation and overlapping function of DnaK and TF in assisting protein folding, the simultaneous deletion of both chaperones causes severe folding defects and aggregation of newly synthesized proteins and is lethal at temperatures above 30°C (Deuerling et al., 1999). Similarly, the function of TF and DnaK can be partially substituted by overexpression of the chaperones GroEL (Vorderwulbecke et al., 2004) as well as SecB (Ullers et al., 2004). Importantly, the extent of exchangeability of chaperones is limited and some nascent proteins require the combined action of TF, DnaK, and GroEL to fold to the native state (Niwa et al., 2012). How the chaperones cooperate and how functional redundancy is conferred is not clear. It also remains open when during translation DnaK, GroEL and others engage their nascent substrates, whether they compete for binding or act simultaneously and how the limited availability of chaperones under conditions of stress can be compensated by other constituents of the network. It is also possible that other chaperones participate in the co-translational network. In eukaryotic organisms nascent chains may be guided by Hsp90 (Geller et al., 2018; Savitski et al., 2018) as well as specialized chaperones (Monkemeyer et al., 2019).

## Cotranslational Formation of Protein Complexes

**FIGURE 3:**
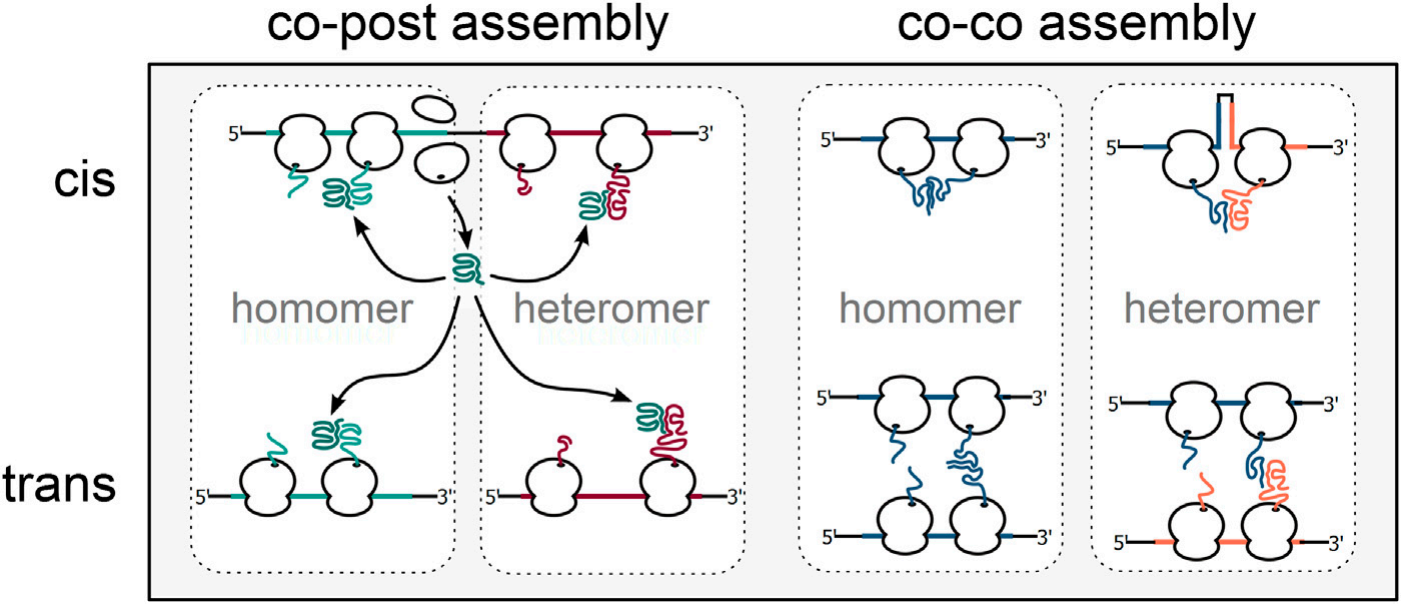
Alternative mechanisms of cotranslational complex assembly. Cotranslational complex assembly can either involve one fully synthesised subunit engaging its nascent interaction partner (co-post assembly, left) or two nascent interaction partners (co-co assembly, right). In bacteria, homomeric as well as heteromeric complexes may be cotranslationally formed between subunits translated from the same (assembly in cis) or separate mRNA molecules (assembly in trans).

About 65% of the bacterial proteome is organized in multi-protein complexes (Hu et al., 2009; Reid et al., 2010; Lynch, 2012). The need to productively form protein oligomers in the highly crowded environment of the cell adds an additional layer of complexity to protein biogenesis. Complex formation was believed to occur post-translationally, driven by diffusion and collision of complex subunits. However, orphan subunits expose hydrophobic interaction interfaces, which enhances unspecific interactions with other macromolecules and can eventually lead to their degradation by the cellular quality control machinery [reviewed in (Juszkiewicz and Hegde, 2018)]. One strategy to cope with this challenge is to initiate assembly cotranslationally [reviewed in (Natan et al., 2017; Williams and Dichtl, 2018; Kramer et al., 2019; Schwarz and Beck, 2019)]. First, yet indirect evidence for the cotranslational assembly of the homo-tetrameric β-galactosidase was already presented in 1963 by David Zipser, who detected β-galactosidase activity in polysome fractions of *E. coli* cell lysates (Zipser, 1963). Recent research demonstrated that cotranslational complex assembly is a universal mechanism (Table 1) and a systematic analysis in yeast found that isolation of 12 out of 31 (∼38%) protein complex subunits led to the copurification of mRNAs encoding their respective interaction partners (Duncan and Mata, 2011), indicating that cotranslational assembly is widespread. Two main modes of cotranslational complex assembly can be distinguished, based on the synthesis state of the interaction partners. One mode is the assembly of a nascent and one fully synthesized polypeptide, recently termed co-post assembly (Bertolini et al., 2021). The alternative mode, termed co-co assembly, involves the interaction of two nascent chains (Figure 3).

**TABLE 1:** An overview of reports on cotranslational complex assembly (adapted from Williams and Dichtl, 2018).

Complex	Organism	Year	Reference
Beta-galactosidase	Bacteria	1963	Zipser (1963)
Immunoglobulin	Metazoa	1979	Bergman and Kuehl (1979)
Myosin heavy chain	Metazoa	1987	Isaacs and Fulton (1987)
Tenascin intermediate filament	Metazoa	1995	Redick (1995)
Reovirus cell attachment protein 1	Eukaryote virus	1996	Leone et al. (1996)
D1 protein of photosystem II	Plants	1999	Zhang et al. (1999)
NF-kappaB1 p50 subunit	Metazoa	2000	Lin et al. (2000)
Voltage-gated K+ channel	Metazoa	2001	Lu et al. (2001)
p53	Metazoa	2002	Nicholls et al. (2002)
IgE high-affinity receptor	Metazoa	2005	Fiebiger et al. (2005)
Periferin	Metazoa	2006	Chang et al. (2006)
Set1C	Funghi	2009	Halbach et al. (2009)
Several *S. pombe* proteins	Funghi	2011	Duncan and Mata (2011)
Luciferase	Bacteria	2015	Shieh et al. (2015)
hERG ion channel	Metazoa	2016	Liu et al. (2016)
SAGA histone acetyltransferase	Funghi	2017	Kassem et al. (2017)
Several *S. cerevisiae* proteins	Funghi	2018	Shiber et al. (2018)
Proteasome subunits Rpt1 Rpt2	Funghi	2019	Panasenko et al. (2019)
TFIID, TREX-2 and SAGA	Metazoa	2019	Kamenova et al. (2019)
*ZNF277–uS5*	Metazoa	2019	Dionne et al. (2019)
Initiation factor complexes	Funghi	2020	Wagner et al. (2020)
>800 cytonuclear proteins	Metazoa	2021	Bertolini et al. (2021)

Extensive studies on the folding and assembly of the bacterial luciferase complex LuxA-LuxB showed that in the absence of LuxA, LuxB assembles into kinetically trapped homodimers (Sinclair et al., 1994) and suggested that the folding pathway of one subunit may be modified by the assembly with its interaction partner (Waddle et al., 1987; Sinclair et al., 1993). More recently, a study based on selective ribosome profiling directly showed that LuxA-LuxB formation occurs by co-post assembly, mainly *via* fully synthesized LuxA engaging nascent LuxB (Shieh et al., 2015). Indicating that uni-directional assembly is the predominant mechanism in co-post assembly, six out of nine complexes analyzed in yeast in a similar study follow a uni-directional assembly mode (Shiber et al., 2018). The sequential assembly order is imposed by the folding properties of the cotranslationally engaged nascent subunits, which are often unstable and get degraded if assembly fails (Halbach et al., 2009; Shiber et al., 2018; Kamenova et al., 2019). Notably, the assembly order of the bacterial LuxA-LuxB reflects the arrangement of the *lux* operon, which is in line with an evolutionary selection for imprinting the order of assembly in the structure of operons (Marsh et al., 2013; Wells et al., 2016). Disrupting the *lux* operon by placing both genes separately at different genomic loci reduced the formation of active luciferase complexes, suggesting that nearby synthesis of subunits on a polycistronic mRNA (cis-assembly) enhances the assembly efficiency. Supporting the notion that co-localized synthesis is a universally employed mechanism, mRNAs encoding the cotranslationally assembling proteasome subunits Rpt1 and Rpt2 are colocalized in yeast, where polycistronic mRNAs are a rare exception (Panasenko et al., 2019). The interaction domains of nascent subunits are often bound by chaperones until the assembly onset (Shieh et al., 2015; Shiber et al., 2018). In bacteria, TF suppresses interactions of nascent LuxA and prevents the premature association of LuxA with nascent LuxB until the complete dimer interface has emerged from the ribosome (Shieh et al., 2015). The general importance of TF in coordinating protein complex assembly is suggested by earlier findings that TF binds a set of fully synthesized proteins, enriched in members of protein complexes, including the ribosomal protein uS7 (Martinez-Hackert and Hendrickson, 2009). A crystal structure showed that a TF dimer encapsulates fully synthesized uS7 in a native-like conformation, masking the contact sites of uS7 to the 16S rRNA in the final 30S assembly. Notably, a TF deletion resulted in a mild ribosome assembly defect under heat stress, supporting the proposed function of TF in complex assembly. Cotranslational complex assembly, on the other hand, might reduce the load for the chaperone system, by establishing crucial interactions early during synthesis and thereby shielding subunits from non-productive interactions. Considering the prevalence of co-post assembly in yeast (Duncan and Mata, 2011) and the fact that bacterial complex subunits are often encoded in operons and translated in close proximity from polycistronic mRNAs, we expect that co-post assembly is also a frequent assembly pathway in bacteria.

Using a ribosome profiling-based method, a recent study showed that also the alternative cotranslational assembly mode, co-co assembly, is a prevalent mechanism employed for the assembly of many homomeric protein complexes in human cells (Bertolini et al., 2021). The study presented evidence that co-co assembly promotes the isoform-specific formation of homomeric complexes, an effect that was previously suggested to mitigate the impact of dominant-negative mutations in the tumor suppressor p53 (Nicholls et al., 2002). Importantly, co-co assembly of human lamins could be recapitulated by heterologous expression in *E. coli*, indicating that co-co assembly is compatible with bacterial translation and the chaperone machineries and may be employed to assemble bacterial protein complexes. Co-co assembly may be mostly employed to assemble homomers with N-terminal oligomerization domains, presumably by the interaction of nascent proteins synthesized by nearby ribosomes on the same mRNA (Bertolini et al., 2021). Ensuring efficient, isoform-specific interactions might in fact be a primary function of co-co assembly. By avoiding the risk of forming chimeric complexes of proteins with similar oligomerization domains co-co assembly could have enabled the reuse of oligomerization domains during evolution (Nepomnyachiy et al., 2017), and the isoform-specific assembly of splice variants in eukaryotes. However, in the context of a polycistronic mRNA, co-co assembly may even facilitate interactions of nascent chains translated from different cistrons and thus the formation of heteromeric complexes.

## Summary and Outlooks

Robust protein synthesis is facilitated by an intricate interplay of all components of the protein synthesis machinery. The system is coordinated at multiple levels, starting from 1) mRNAs, that contain information that guides translation elongation rates of ribosomes to control protein folding and also warrant the colocalized synthesis of cotranslationally interacting protein subunits, 2) sequence and structural features of nascent chains that facilitate the binding of enzymes, targeting factors and assembling subunits, and 3) the crosstalk between ribosomes, nascent chains and maturation factors. While we have made significant progress in understanding some of the general principles that guide this process, detailed knowledge of the molecular mechanisms is still rather limited and many open questions remain. How do ribosomes sense the folding state of nascent chains and the status of their interactions with chaperones and protein complex subunits and is this feedback mechanism widely used by nascent chains to control their cotranslational maturation? How do chaperones determine cotranslational substrates and affect their conformation and how are the cotranslational activities coordinated between chaperones? Finally, we need to obtain information on the prevalence and the mechanisms guiding the cotranslational assembly of protein complexes. It will be fascinating to see whether also periplasmic and membrane proteins assemble cotranslationally and how the assembly of all classes of proteins might be coordinated by the action of chaperones, targeting factors and the translating ribosome. Furthermore, gaining insight into the folding state of nascent subunits will be crucial to understand how structural features determine assembly processes. Answering these questions is a formidable task and will require the contribution of multiple disciplines of basic research.
